# EBV lymphoproliferative-associated disease and primary cardiac T-cell lymphoma in a STK4 deficient patient: A case report

**DOI:** 10.1097/MD.0000000000008852

**Published:** 2017-12-01

**Authors:** Roya Sherkat, Mohammad Reza Sabri, Bahar Dehghan, Hamid Bigdelian, Nahid Reisi, Nooshin Afsharmoghadam, Hamid Rahimi, Narges Rahmanian, Cristoph Klein

**Affiliations:** a Acquired Immunodeficiency Research Center; b Pediatric Cardiovascular Research Center, Isfahan Cardiovascular Research Institute; c Department of Cardiovascular Surgery; d Department of Pathology, Isfahan University of Medical Sciences, Isfahan, Iran; e Department of Pediatrics, Dr. von Hauner Children's Hospital, Ludwig Maximilians University, Munich, Germany.

**Keywords:** primary cardiac lymphoma, primary immunodeficiency, STK4 deficiency

## Abstract

**Rationale:**

Primary cardiac lymphoma (PLC) is an extremely uncommon malignancy. PCL is more common in secondary immunodeficient patients. In this report, we describe a unique case of PLC who had been diagnosed as a STK4 deficient patient. This case is the first Primary immunodeficiency (PID) patient developing PCL in the world.

**Patient concerns:**

An eleven-year-old girl, a known case of PID, was referred to the pediatric cardiology department because of chest pain and dyspnea. Her CXR revealed cardiomegaly without mediastinal involvement and the echocardiography showed a mild pericardial effusion and cystic-shape echogenic masses.

**Diagnoses:**

After a period of missed follow up, she presented with respiratory distress following with syncope at the clinic because of a pressure effect of a large mass on the right ventricular outflow tract (RVOT) .An emergency operation was done for debulking of the tumors and resolving of RVOT obstruction. Biopsy and immunohistochemical staining was revealing “T-cell lymphoma”, non-Hodgkin's type.

**Interventions:**

Chemotherapy was done with cyclophosphamide, methotrexate, adriamycine, vincristine, hydrocortisone and allopurinol.

**Outcomes:**

The tumors shrank after chemotherapy initiation and she stayed stable for almost one month. Finally, she developed sever thrombocytopenia during her chemotherapy and died because of lung hemorrhage two months after her operation.

**Lessons:**

Although PCL is very rare, it must be considered in the differential diagnosis of intracardiac mass or refractory pericardial effusions, especially in PIDs which are widely known for developing EBV-associated diseases such as lymphoma.

## 1 Introduction

Primary immunodeficiency (PID) patients are at great risk of malignancy in comparison with the normal population. After infections, malignancy is the second cause of death in these patients.^[1]^ The most widely recognized sorts of malignancies among PID patients are non-Hodgkin lymphoma (NHL), which represent 48.6% of tumors found in PID patients.^[2]^ NHL developing is frequently associated with the Epstein-Barr virus (EBV). In fact EBV infection is a potentially premalignant incident in PID patients.^[3]^ B-cells infected by EBV stimulate a notable activation of immunoregulatory T cells. Therefore, a restoration of immune homeostasis during convalescence is required. This homeostasis is not completely reached in a person with significant immune deficiency.^[4]^ Consequently, immunodeficient patients infected by EBV with impairment of lymphocytic cytotoxic pathway, T-cell signaling, or T-B cell interaction are at great risk of developing immunoregulatory disturbances and lymphoproliferative diseases such as lymphoma.^[5,6]^

Primary cardiac lymphoma (PLC) is a very rare malignancy (under 200 cases), occurring more frequently in secondary immunocompromised patients.^[7,8]^ PCL is typically of a non-Hodgkin type, and involves just the heart or pericardium without confirmation of extracardiac involvement. PLC represents around 1% of the primary cardiac tumors and 0.5% of the extranodal lymphomas. Most PCLs are of B-cell origin and PCLs with T-cell origin are extremely rare. Moreover, PCL is very rare in children.

In this report, we describe a unique case of primary cardiac T-cell lymphoma with PID who has been diagnosed as a novel PID syndrome, STK4 deficiency. STK4 protein is critical for maintenance of lymphocytes and control of unrestricted EBV-induced lymphoproliferation. Human STK4 deficiency afford a PID syndrome influencing T cells, B cells, and potentially neutrophil granulocytes.^[9]^ To the best of our knowledge, this case is the first PID patient developing PCL and the first known little girl with primary cardiac T-cell lymphoma in the world.

## 2 Case report

**Figure 1:**
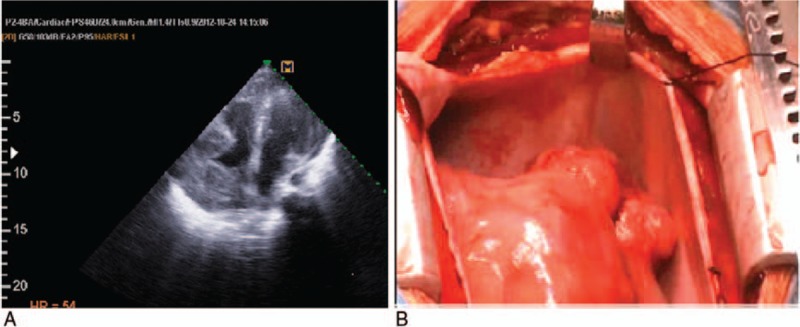
(A) Transthoracic echocardiography, 2 echogenic masses with lucent centers on RA free wall and RVOT. (B) Two large extra cardiac masses with pressure effect on RVOT.

**Figure 2:**
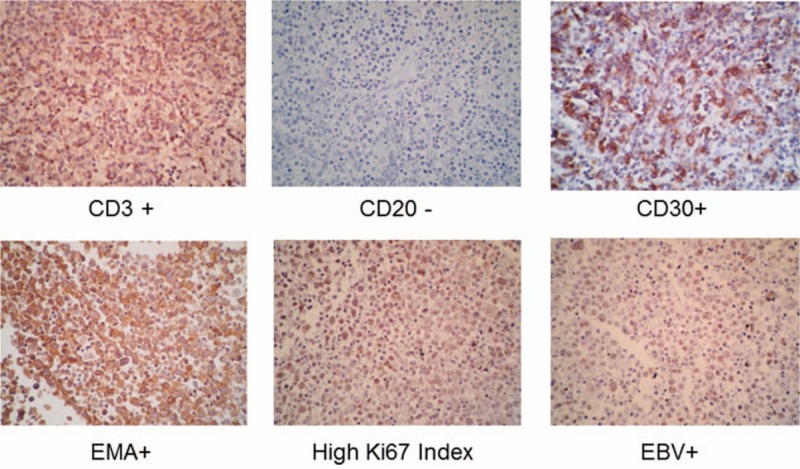
Biopsy and immunohistochemical staining of cardiac mass.

An 11-year-old girl from a consanguine family died because of primary cardiac T-cell lymphoma. She was a known case of PID. Her genetic background had been investigated in 2010 at Germany and for the first time a novel PID syndrome caused by a homozygous nonsense mutation in STK4 was found in this family (the patient, her aunt, and her uncle).^[9]^ Human STK4 deficiency causes a PID syndrome affecting T cells, B cells, and possibly neutrophil granulocytes. She had history of recurrent bacterial infections, viral infections, mucocutaneous candidiasis, cutaneous warts, mouth ulcers, skin abscesses, recurrent sinusitis and rhinitis, and at least 2 episodes of staphylococcal pneumonia. Her hematological investigations revealed T- and B-cell lymphopenia and intermittent neutropenia. Her echocardiogram was revealed structural cardiac abnormalities, including atrial septal defect. At the age of 9, she developed generalized lymphadenopathy and chronic fever. Her cell count showed significant increase in CD8^+^ T-cells and NK cells, a relative increase in transitional B cells and evidence of hypergammaglobulinemia. The lymph node biopsy showed an EBV-associated B-lymphoproliferative disorder with plasmacytoid differentiation, κ-light chain restriction, monoclonal immunoglobulin gene rearrangement and positive EBV-DNA PCR of tissue biopsy. Epstein-Barr encoding region (EBER) in situ hybridization proved EBV infection. Three years after generalized lymphadenopathy she was referred to the pediatric cardiology department because of chest pain and dyspnea. Her CXR revealed cardiomegaly without mediastinal involvement and the echocardiography showed a mild pericardial effusion. During follow-up her echocardiograms showed 3 cystic-shape echogenic masses on the inter-atrial septum, RV free wall, and inter ventricular septum on and off. After a period of missed follow-up, she presented with respiratory distress followed with syncope at the clinic because of a pressure effect of a large mass on the right ventricular outflow tract (RVOT) (Fig. 1A). It is necessary to state that informed consent was obtained for all photographic documentation. An emergency operation was done for debulking of the tumors and resolving of RVOT obstruction. During the operation, there were at least 2 large masses in the RVOT and right atrium. It was not possible to remove the tumor masses, so a Glenn shunt was inserted for compensation of RVOT obstruction (Fig. 1B). Biopsy and immunohistochemical staining was positive for CD30, Ki67, EBV, and EMA and negative for CD20 revealing “T-cell lymphoma,” non-Hodgkin type (Fig. 2). Chemotherapy was done with cyclophosphamide, methotrexate, adriamycin, vincristine, hydrocortisone, and allopurinol. The tumors shrank after chemotherapy initiation and she stayed stable for almost 1 month.

Nevertheless, her malignancy invaded to the lungs and the liver. She had pleural effusion with infiltrations of malignant cells and severe lung infection due to the neutropenia. She also developed attacks of generalized tonic-clonic seizures due to brain sinuses thrombosis. Finally, she developed sever thrombocytopenia during her chemotherapy and died because of lung hemorrhage 2 months after her operation

## 3 Discussion

In this report, we describe a unique case of primary cardiac T-cell lymphoma with STK4 deficiency, a PID syndrome. PLC is defined as a typical non-Hodgkin type and involving just the heart or pericardium without confirmation of extracardiac involvement. PCL is a very rare malignancy occurring more frequently in secondary immunocompromised patients. PCL represents around 1% of the primary cardiac tumors and 0.5% of the extranodal lymphomas. Most PCLs are of B-cell origin, while PCLs with T-cell origin are extremely rare.^[7,8]^ The male-to-female ratio is 1.94, with a median age of 63. Moreover, PCL is very rare in children. The most common symptoms and signs are dyspnea, chest pain, pericardial effusion, heart failure, and AV-block.^[10]^ A careful work-up ought to incorporate transthoracic and transesophageal echocardiography, computed tomography, and magnetic resonance imaging. The ECG shows nonspecific change and is non-valuable. Chest radiography reveals nonspecific findings, such as a cardiomegaly or pleural effusion. PCL diagnosis is confirmed by histology/cytology.^[11–13]^

The clinical manifestations of PCL are all nonspecific making early diagnosis troublesome. Unlike the other cardiac tumors PCL is rapidly progressive and fatal though; a physician should always keep in mind PCL as a differential diagnosis in cardiac mass or refractory pericardial effusion especially in immunocompromised patients. These tumors often result in a clinical emergency requiring immediate diagnosis and instant treatment. PCL should be treated as soon as possible because of the possibility of sudden death caused by disturbance of hemodynamics, ventricular fibrillation, or massive pulmonary embolism. The diagnostic lag leads to a poor prognosis in PCL.^[7,14,15]^ Chemotherapy is the main successful treatment for PCL and some patients display a clinical remission or cure (radiotherapy does not appear to enhance quiet survival rate, and a radical surgical methodology is not prescribed). In any case, PCL with T cell origin is resistant to chemotherapy, which makes it more aggressive with a shorter survival time.^[16]^ The survival is usually <1 month without treatment but has been extended up to 5 years by palliative treatments in selected cases. Autologous stem cell transplantation is a promising treatment in resistant cases.

As we mentioned before, PCL is a NHL. NHL happens more frequently in immunocompromised patients and is frequently caused by EBV infection.^[7,10]^ EBV infection is a potentially premalignant incident in PID patients.^[3]^ Some kinds of primary immunodeficiencies (PIDs) are widely known for developing EBV-associated disease. The EBV can cause a self-limiting lymphoproliferative disorder, called infectious mononucleosis (IM). However, EBV infection rarely results in severe, often fatal conditions, including lethal IM, hemophagocytic syndrome, polyclonal lymphoproliferative disorders, and malignant lymphoma. PIDs with lymphocyte cytotoxic pathway impairment or T-cell dysfunction are at great risk for developing these fatal conditions.^[17]^

Human STK4 deficiency affords a PID syndrome influencing T cells, B cells, and potentially neutrophil granulocytes. Clinically, the patients shared intermittent neutropenia, T- and B-cell lymphopenia, atrial septal defect, recurrent viral and bacterial infection, mucocutaneous candidiasis, cutaneous warts, and skin abscesses.^[9]^ In 50% of STK4 deficiency reported cases persistent EBV viremia and EBV-associated lymphoproliferative disease has been reported.^[18]^

Natural killer (NK) cells and EBV-specific cytotoxic CD8+ T lymphocytes (CTLs) are essential for the elimination of virus-infected cells and surveillance against tumor cells. B-cells infected by EBV stimulate a notable activation of immunoregulatory T-cells. Therefore, a restoration of immune homeostasis during convalescence is required. STK4 protein is critical for maintenance of lymphocytes and control of unrestricted EBV-induced lymphoproliferation. Indeed STK4 is necessary for activation of FOXO1 and FOXO3, crucial transcription factors for powerful CTL response to chronic viral infection and T cell homeostasis. Though, this homeostasis is not completely reached in a person with STK4 deficiency.^[9,19–21]^ Impairment of CTLs and NK cells cytotoxic activity impede efficient removal of infected cells leading to continuous antigenic stimulation. This condition precludes downregulation of the elicited immune response resulting in persistent hyperactivation and proliferation of these effector cells.^[18]^ Consequently, immunodeficient patients with impairment of lymphocytic cytotoxic pathway, T cell signaling or T-B cell interaction which is infected by EBV are at great risk of developing immunoregulatory disturbances and lymphoproliferative diseases such as lymphoma.^[5,6]^

This patient had STK4 deficiency and was susceptible to develop EBV-associated disease but because of the rare incidence of PCL especially in children, her presentation was unexpected. Clinicians should always keep in mind EBV-associated lymphoproliferative disease in PID patient with EBV infection history especially those who have shown reduced number or function impairment of NK cells or CTLs.

## 4 Conclusion

Malignancy is the second most common cause of death in PID patients and the most common type of malignancy among these patients is NHL. PLC is a very rare malignancy, which is usually non-Hodgkin type. PLC has often poor prognosis, mainly because of delay in the diagnosis, owing to a lack of clinical suspicion of this rare cardiac malignancy. PCL may cause sudden death due to disturbance of hemodynamics, ventricular fibrillation, or massive pulmonary embolism. However, early diagnosis and initiation of appropriate chemotherapy may result in a remission of disease and survival increase. Although PCL is very rare, it must be considered in the differential diagnosis of intracardiac mass or refractory pericardial effusions, especially in PIDs which are widely known for developing EBV-associated diseases such as lymphoma.
